# Vibration-Based Diagnostics of Radial Clearances and Bolts Loosening in the Bearing Supports of the Heavy-Duty Gearboxes

**DOI:** 10.3390/s20247284

**Published:** 2020-12-18

**Authors:** Pavlo Krot, Volodymyr Korennoi, Radoslaw Zimroz

**Affiliations:** 1 Faculty of Geoengineering, Mining and Geology, Wroclaw University of Science and Technology, 50-421 Wroclaw, Poland; radoslaw.zimroz@pwr.edu.pl; 2 Z.I. Nekrasov Iron and Steel Institute of National Academy of Sciences of Ukraine, 49050 Dnipro, Ukraine; koren_noy@ukr.net

**Keywords:** radial clearance, bearings, bolts loosening, nonlinear model, transient vibration, condition monitoring

## Abstract

The problem solved in this research is the diagnosis of the radial clearances in bearing supports and the loosening of fastening bolts due to their plastic elongation (creep) or weak tightening using vibration signals. This is an important issue for the maintenance of the heavy-duty gearboxes of powerful mining machines and rolling mills working in non-stationary regimes. Based on a comprehensive overview of bolted joint diagnostic methods, a solution to this problem based on a developed nonlinear dynamical model of bearing supports is proposed. Diagnostic rules are developed by comparing the changes of natural frequency and its harmonics, the amplitudes and phases of shaft transient oscillations. Then, the vibration signals are measured on real gearboxes while the torque is increasing in the transmission during several series of industrial trials under changing bearings and bolts conditions. In parallel, dynamical torque is measured and its interrelation with vibration is determined. It is concluded that the radial clearances are the most influencing factors among the failure parameters in heavy-duty gearboxes of industrial machines working under impulsive and step-like loading. The developed diagnostics algorithm allows condition monitoring of bearings and fastening bolts, allowing one to undertake timely maintenance actions to prevent failures.

## 1. Introduction

The rolling (ball, roller) and journal bearings have a wide implementation in numerous industrial machines. Although bearings are the most standardized elements of transmissions, they are probably the most difficult to tune elements in the assembly units. Together with rotating shafts, bearings constitute a complex thermo-mechanical system, which performance greatly depends on the radial and axial clearances. Any deviations of ring position caused by improper installation or shaft misalignment, or violation of shaft diameter tolerances may interrupt foreseen lubrication flows and homogeneous cooling that in its turn provokes thermal deformations and reduction of bearing life. In addition, clearances may cause excessive dynamical loading in the case of gaps re-opening under non-stationary or reverse loading of a machine’s driveline.

**Figure 1:**
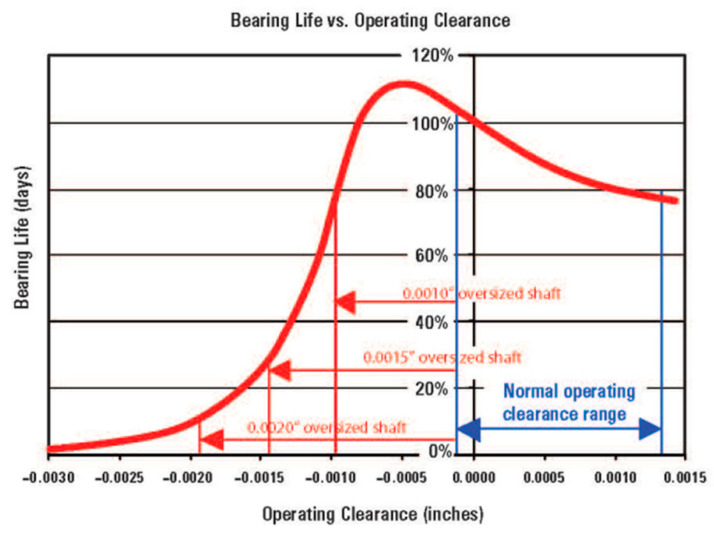
Dependence of the bearing life on the operating clearance [1].

Therefore, clearances may be considered as the most important operational parameters of drivelines because of their direct influence on bearing life and the overall reliability of rotating machines. Every producer of rolling bearings has a specification for axial and radial clearances. Neglecting the recommended maintenance rules, installation procedure, revision schedule, lubricant type and its supply rate cause the majority of failures, even more so than external loading. The influence of an oversized shaft diameter on the bearing life taken from work [1] is shown in Figure 1. This deviation in size can be caused, e.g., by heating of shaft itself or the bearings.

As it follows from the ISO Standard 5753-1981 (ANSI/AFBMA Standard 20), limits for clearances in the unmounted radial ball and roller bearings are categorised into five “Classes” or “Groups”, which are denoted as “Normal,” “greater than Normal,” (C3 suffix), “less than Normal” (C2) [2]. Class C3 is used to avoid too little internal bearing clearance in machine operation and applied for mining and metallurgical machines.

One of the most important factors determining accurate clearances in bearings are the bolted joints, which fasten the shaft and the bearings covers to the gearbox housing. The fundamental principle of bolted joints assumes that the designers of machines should predict their operational loads and avoid opening of any joint contacted surfaces when forces are greatly increasing and additional components of stresses (torsional, bending, shear) appear in the bolt shank [3]. The greater the preloading (limited by yield stress), the less chance of bolt failure. Although bolted joints preloading has great importance, their tightening torque is not always available for control during repairs and shafts replacement in the transmissions, especially for large-scale industrial plants where bolt diameters can reach 100–150 mm and even more.

The metric bolts for heavy-duty gearboxes should have property classes 10.9 or 12.9 (ASTM F568M) and are made of alloy steels (see Table 1).

The strength and durability of high-grade bolt steels (40Cr, 30CrMnSi) are usually improved by quenching and tempering. Application of deep cryogenic treatment is also available like in other important parts of industrial plants [4], but rarely used in practice. An over-elastic pre-load of the screw-nut pair allows one to obtain a significantly higher load capacity, and a self-forming screw is proposed in this regard in [5] to achieve residual compressive stress at the thread root.

However, the bolts of heavy-duty gearboxes of mining and metallurgical machines are subjected to fatigue damage due to frequent overloading beyond the allowed yield limits of steel parts.

### 1.1. Dynamical Models of Gearbox Shafts and Bearing Supports

In the dynamics of rotating machines, the complex system of “rotor-bearing-housing” is commonly considered to reflect the existing internal relations and coupling effects of natural modes. Radial clearance and waviness of bearings are investigated in a 4-DOF model in [6]. A study of the effect of the radial internal clearance of a ball bearing on the dynamics of a rotor is given in [7]. Many studies are conducted on regular and chaotic dynamics and stability regimes estimation under the action of imbalances, external and parametric excitation. These studies of heavy rotor and bearings interaction dynamics are related to constant conditions of speed and loading, mainly found in power generation units. Transient resonances under gradually changing rotation speed are also investigated using parameters maps and Campbell diagrams. Nonlinear vibration of a two-DOF rotor supported by rolling bearings with clearance is investigated in [8] where the inner and the outer race centres are assumed to not be collinear. As noted in [9], all mining machines are operated in dusty environments causing quick contamination of any lubricant by hard sharp particles and resulting in the wear of bearings and inner gearbox shaft and gear misalignment (IGSGM), which is difficult to account for in gearbox modelling [10] under non-stationary loading conditions. Therefore, transient vibrations of shafts in bearing supports are less investigated, e.g., in pinion stand [11] and gearboxes [12]. The effect of bolt loosening on the torsional dynamics during the operation of machines in industrial plants is investigated in [13] and split path gearbox dynamics with parametrical excitation are represented in [14]. An advanced 16-DOF dynamical model of a gearbox with radial clearances in the bearings is developed and simulated in [15], where the authors noted that bearings contribute to natural modes of vibration.

To understand the interaction of bolted joints parts, a simulation of a 3D finite-element model was conducted in [16] and the authors concluded that a creep slip phenomenon exists at the contact surface, which causes bolt self-loosening. Other different effects which influence self-loosening of bolted joints have been investigated, e.g., the repetition of small slippages at the bearing surface [17] and the effect of hole clearance and thread fit [18]. Transverse displacements and shear stress are considered as the main factors of self-loosening, hence, friction forces created by pretension forces in a thread and joined contacts are the main remedy against it. Usually, bolts are prevented from loosening by different methods, e.g., Grover washers, split-pins, second nuts. However, reliable fixing does not eliminate the creep and plastic deformation of bolts and threaded studs under severe axial loading. Details of joints simulation are presented in the book [19]. Dynamic shear stress represents the main contribution to self-loosening of bolted joints [20]. The authors discovered that there is a critical shear load amplitude, below which loosening would not happen and bolted joints made of quenched and tempered steel and stainless steel have a significant anti-loosening performance.

### 1.2. Diagnostics of Bearing Supports and Bolts Loosening

The diagnosis of bolts loosening, not only in gearboxes but also in structural elements of buildings or bridges is a complicated scientific and engineering task taking into account the potentially huge number of bolted joints and the dramatic consequences of their loosening which leads to unpredicted redistribution of internal loads between other bolts in joints.

There are several approaches to health monitoring of bolted joints. They can be classified into two big groups: based on the use of local sensors or “smart bolts” and the modal analysis of the whole structure or machine. Local contact surface sensors, e.g., by electrical conductivity [21], tension stress measurement by ultrasonic waves [22,23] high-frequency acoustic emission of low-speed rotating machinery [24], using high-order harmonics and spectral sidebands [25], wave energy dissipation (WED) and vibroacoustic modulation (VM) [26,27,28]. Sensing instrumentation also includes piezoelectric active sensing [29,30,31,32,33] using wearable sensors [34] and smart washers [35,36,37,38,39] based on lead zirconate titanate (PZT) transducers, which generate testing stress waves as the actuators. These methods can be supported by wireless data transfer technologies and remote data accumulation for critical large-scale civil and industrial structures.

The axial force reduction decreases the stiffness of the “bolt-nut” assembly leading to a shift in the characteristic peak frequency of the bending mode. A generation of acoustic emissions occurs due to the relative movement between the contacting elements of joints, e.g., the bolt shank within the clearance hole. Elongation of the bolt due to tensile stress is defined by the time-of-flight (TOF) of ultrasonic waves. The reliability of such methods may be enough high under laboratory conditions but they require more investments for additional sensors and their implementation in actual mining and metallurgical machines is complicated.

In recent years, visual image processing methods have gained a popularity in bolts loosening diagnostics [40,41,42] using deep learning techniques [43,44,45], Hough transforms [46,47], support vector machine (SVM) [48] density-based spatial clustering of applications with noise (DBSCAN) [49], empirical mode decomposition–based nonlinear system identification [50] and convolutional neural networks (CNNs) [51]. A brief review of bolted joint monitoring is given in [52]. This subclass of methods allows one to increase the productivity of scheduled revisions on large-scale structures even in automatic mode by flying mobile drones equipped with cameras and embedded image processing methods. Reliability of such methods is greatly dependent on previous revisions, which are used as the reference points for nuts’ position change detection. However, these methods cannot detect bolts creep or axial deformations if not accompanied by the nut or head shifting.

Another class of bolts loosening diagnostic methods uses system modal parameters. The decrease of bolts tightening influences various boundary conditions and can be detected by the changes in natural frequency, phase shift and modal damping [53] due to less contact friction or stiffness. For example, vibration transmissibility function is determined as a more reliable parameter to identify the state of the joint while natural frequency and modal damping in lower modes appeared less reliable. Strain measurements are carried out using fibre Bragg-grating (FBG) sensors [54,55] and optimal tightening sequence is determined in multi-bolt connections [56]. In other studies [57,58,59,60], theoretical and experimental studies have been conducted for an assessment of modal properties and frequency response functions applicability for detection of structural bolted joint degradation [61].

Some methods of radial clearance diagnostics by vibration signals are given in [62,63,64,65,66,67,68,69,70,71,72,73]. The effect of rolling bearing clearances on diagnostic signatures is investigated in [65] in the 5-DOF nonlinear dynamic model, which imitates gradual wear of bearings, using an experimental identification method based on FRF decoupling and optimization [66]. Instrumentation for angular backlashes diagnostic in drivelines by shaft rotations sensors is developed in [74].

A truck transmission gearbox FEA simulation in [75] showed that the loosening of one bolt varies the natural frequency in the 1637–2674 Hz range. Experimental research conducted in [76] on the flange plates joints of a wind turbine tower showed that the first-order phase difference parameter is more sensitive to the looseness of the bolts. A combination of FEA with vibration and impedance responses measurement of wind turbine tower for bolts loosening detection is given in [77]. FEM simulation is used in [78] for a lightning rod flange-bolt structure unit (FBSU) analysis. The authors in [79], considering a bolt as an axially stressed and clamped at both ends beam, proposed a simple method to determine the bolt tightness by natural frequencies and damping ratios in hammer impact tests, in particular, the first transverse natural frequency [80]. Damping ratios and nonlinearity in the bolt’s frequency response were examined in [81], and the instantaneous natural frequencies are found to be practically independent of the amplitude of hammer test impacts.

The estimation of a residual lifetime of centrifugal pumps is conducted in [82] by FEM simulation taking into account the tightening of bolted connections, technological and temperature loads with a corresponding thinning of the housing walls. In [83], a tightened bolt is modelled as a plane beam having two linear end springs (transverse and rotational). A more general multi-degree-of-freedom (MDOF) model is developed in [84] accounting not only for bolt tightening but material or boundary nonlinearities in structures and faults are also detected with a second-order output spectrum (SOOS) and local tuning approach (LTA). A comprehensive overview of bolt tightening force measurement and loosening detection is presented in [85] where the authors considered different methods and corresponding instrumentation. The authors in [86] demonstrated the implementation of an electronic stethoscope and a continuous wavelet transform (CWT) technique to process and display the transient responses of a bolted joint in a structure to detect loosening and enhance audible perception using audio output. In [87] the authors used instantaneous angular speed (IAS) measurements to analyse the size of local defects in bearings with clearance, which is accompanied by transient signals when entering and exiting the damaged zone. Another example of bearing spalling defect diagnostics is given in [88], where the authors estimate the duration of transient signals at the natural frequency caused by stiffness variation of the structure. The problem is that numerous tightenings using uncontrolled torques make the distinction between elastic and plastic elongation difficult for a bolt of large diameter, while tolerance temperature compensation is required in assemblies of heavy-duty gearboxes of mining and metallurgical machines working under harsh conditions.

The majority of known vibration-based diagnostic methods are based on time, frequency or time-frequency domains analyses at the kinematic frequencies of the bearings and oriented towards detection of local damage on rings, separators and rolling elements. Methods of radial clearance estimation in bearings are less developed. In general, gaps in the shaft supports can be determined by the amplitude change of the vibration spectrum or the envelope (Hilbert-Huang transformation) of the filtered signal at the kinematic frequencies of a mechanism by the presence of harmonics of half the rotational frequency of the rotor. Noteworthily, the RMS values of vibrations across the whole frequency band are not standardized for such machines. Some methods are accommodated to bearing condition monitoring under non-stationary working conditions [89,90,91,92,93] using wavelets, bicoherence, spectral kurtosis and covariance, and high-order spectrum techniques to detect non-linear features of the signals. Methods are developed for impulsive components extraction in the presence of non-Gaussian heavy-tailed noise due to stochastic impacts from mineral pieces [94,95,96,97,98,99,100,101] or transient vibration analysis in industrial rolling mills [102,103].

The accuracy of vibration-based diagnosis depends on the reference values corresponding to a healthy condition. For serial machines, such references and alarm levels are determined by a group of similar mechanisms or on a new machine after running at a constant speed and nominal load level. The unique design of mining and metallurgical machines requires new approaches to determining reference values and the alarm levels of health indicators when bearing wear is critical.

Historically, direct measurements of installation clearances or the full wear (gap) in the elements of bearings in large-scale machines are conducted by lifting of their heavy shafts with special mechanisms [67] or cranes in workshops and then sticking calibrated probes into gaps. These methods require partial disassembly of the machines, e.g., removing the end caps on gearbox shafts. This is time-consuming and contributes to damage of the finished surfaces of the bearings parts that further reduce the total life of the machines.

The scope of this paper covers the development of condition monitoring methods of unique mining machines and rolling mills for their maintenance planning based on the actual condition of the equipment, namely, measurements of wear (radial clearances) of the bearing supports and fastening bolt loosening. Section 2 presents the diagnosis methodology based on a dynamical model of shaft supports. Section 3 gives the results of a dynamical model simulation for different conditions. Section 4 contains the results of industrial trials to verify the developed diagnostic method. Finally, the results of the research are considered in the Discussion and Conclusions sections.

## 2. Methodology of Diagnostics

The essence of the proposed method is as follows. Wear of the bearings in the supports of transmission shafts, such as heavy-duty gearboxes of the mining machines and rolling mills, causes the appearance of radial gaps, which are opened during idling of the machine and closed after any quick loading when the shaft is moving in the supports to its steady working position.

At the initial stage of wear, the open radial gaps cause nonlinearity (such as a dead zone) of the stiffness characteristics in the transmission supports, which leads to a significant increase in the amplitude of shock loads. Under gradual wear, contact opening of the fastening bolted joints occurs under more severe impacts. This leads to a fracture point in the stiffness characteristics of the bearing support. Wear of bolts (creep and plastic elongation) may cause failures of even newly installed gears, which have not yet been subjected to cyclic fatigue, therefore, their diagnostics is an important part of machine maintenance.

### 2.1. Non-Linear Dynamical Model of Bearing Supports

**Figure 2:**
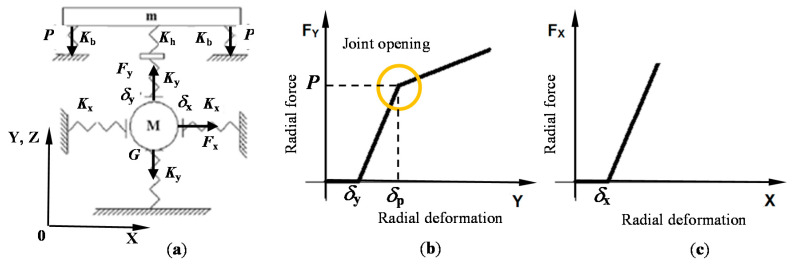
Calculating scheme of the gearbox shaft in the bearing support (**a**); the nonlinear stiffness characteristics of the bearing support with a clearance in vertical (**b**) and horizontal (**c**) direction.

A non-linear dynamical model with a circular clearance is developed to investigate transient vibrations in bearing supports. The disturbance for the shaft comes from the torque applied to the gearbox. The calculating scheme of the dynamical model is shown in Figure 2a, which describes the gearbox shaft oscillations in the bearing support with the initial gap and bolted joint opening in a vertical direction. Notations are as follows: *P*—bolt pre-load (tightening force); *F_x_*, *F_y_*—horizontal and vertical forces; *G* = *M g*—gravity force of shaft; *m*—a mass of the bearing cover; *M*—a mass of the gearbox shaft; *K*_b_—stiffness of fastening bolts; *K*_h_—stiffness of the gearbox housing; *K*_y_—stiffness of the bearing in the vertical direction; *K*_x_—stiffness of the bearing in the horizontal direction; *δ*_y_, *δ*_x_—the total clearance along the vertical axis *Y* and horizontal axis *X*; *δ*_p_—deformation at the fracture point.

The nonlinear (piecewise) stiffness characteristics of the bearing support in the *Y* and *X* directions are given in Figure 2b,c. The *F*_y_ graph has an additional fracture point (*δ*_p_, *P*) and less stiffness beyond it, which corresponds to the bolts loosening effect and contact opening above the certain level of loading. The *F*_x_ graph has no such point because of gearbox housing has no changes in stiffness characteristic after gaps closing in this direction.

The following assumptions are considered in the dynamical model simulations:

- the effect of friction is accounted for in the damping factor;
- the stiffness of bolts and bearings without clearances is independent of load;
- shaft impacts do not produce plastic deformations of bearings and bolts;
- shaft motion is synchronous for both bearing supports.
- in the case of joint opening, vertical shaft motion is without shear stress on bolts.

The system of nonlinear differential equations of the model is as follows:

(1) $$M\ddot{x}+K_{x}x+C_{x}\dot{x}=\left|F_{t}\right|\;\frac{\tan\alpha_{n}}{\cos\beta }$$

(2) $$M\ddot{y}+K_{y}y+C_{y}\dot{y}=F_{t}-G-K_{m}z$$

(3) $$m\ddot{z}+K_{m}z+C_{b}\dot{z}=K_{y}y\;-n\;P-mg$$

where *x*, *y*, *z*—coordinates of motion; *C_x_, C_y_, C_b_*—damping factors; *K_m_*—total stiffness of bolts (*K_b_*) and housing (*K_h_*). The right part in formula (1) is a radial force in helical gearing; *F_t_*—tangential force in the gearing; *α_n_*—normal pressure angle of gear; *β*—helix angle of gear; *n*—number of bolts; *P*—bolt pre-load (tightening force).

Nonlinear stiffness with radial clearance is described as follows:

(4) $$K_{x}=\left\{\begin{array}{cc} K_{x}, & if\;\delta_{x}<\sqrt{x^{2}+y^{2}}\\ 0, & otherwise \end{array}\right\}$$

(5) $$K_{y}=\left\{\begin{array}{cc} K_{y}+K_{m}, & if\;\delta_{y}<\sqrt{x^{2}+y^{2}}\leq \delta_{p}\\ K_{y}+K_{b}, & if\;\sqrt{x^{2}+y^{2}}>\delta_{p}\\ 0, & otherwise \end{array}\right\}$$

The components of deformations projected on axes *X* and *Y*:

(6) $$\Delta_{x}=\frac{x}{\sqrt{x^{2}+y^{2}}}\left(\sqrt{x^{2}+y^{2}}-\delta_{x}\right)$$

(7) $$\Delta_{y}=\frac{y}{\sqrt{x^{2}+y^{2}}}\left(\sqrt{x^{2}+y^{2}}-\delta_{y}\right)$$

where *δ_x_, δ_y_*—initial clearances in the bearing; $\sqrt{x^{2}+y^{2}}$—shaft displacement from the initial position; $\left(\sqrt{x^{2}+y^{2}}-\delta_{x}\right)$—bearing deformation.

The absolute value of the reaction force in the gearbox support is:

(8) $$R=\sqrt{F_{x}^{2}+F_{y}^{2}}$$

where *F_x_*, *F_y_*—horizontal and vertical forces acting on the shaft.

The main mode frequency of shaft free vibrations in the bearings is determined as:

(9) $$f_{n}=\frac{1}{2\pi }\sqrt{\frac{K_{y}+K_{m}}{M}}\;(y\;<\;\delta_{p});\;f_{n}=\frac{1}{2\pi }\sqrt{\frac{K_{y}+K_{b}}{M}}\;(y\;\geq \;\delta_{p})$$

During the period of rising load on the working tool (digging phase of a mining machine, slab biting in rolling mills), the reaction on the bearing of each transmission element depends on the radial gaps (including installation clearances and wear) and bolt loosening (weak tightening). In this case, the greater the radial clearance, the greater the amplitude of the vibration and phase shift at the natural frequency of radial oscillations of the shaft. These features are common for piecewise linear systems and can be used for the diagnostics of bearings and bolted joints.

### 2.2. Dynamical Model Simulations

The parameters for dynamical model simulations are taken from the investigated gearbox specification. Gears meshing angles *α*_n_ = 20°, *β* = 33°, the input shaft mass *M* = 1570 kg; four (2 per side) double row tapered roller bearings 2,097,960 SPZ (300 × 420 × 160 mm); six bolted studs M48 × 800 mm per every support, housing cover mass is *m* = 940 kg. The stiffness of housing *K_h_* = 1.480 × 10^9^ N/m; bearing *K_y_* = *K_x_* = 1.544 × 10^5^ N/m; bolts *K_b_* = 7.509 × 10^8^ N/m. Pre-loading of bolted studs is varied from 20% and up to 70% of the proof stress in the range of elastic deformation of steel.

**Figure 3:**
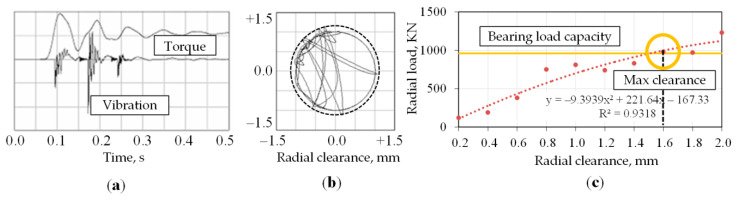
Results of simulations in the time domain: torque and vibration (**a**); trajectory (orbit) of shaft centre of mass motion (**b**); the dependence of maximal radial load in the bearing on radial clearance (**c**).

The solutions for the system of differential Equations (1)–(3) were obtained by the 4th order Runge-Kutta method of numerical integration with a constant time step. Results of the dynamical model simulations are shown in Figure 3. Time series of torque and vibration show similar behaviour (see Figure 3a) because the increase of torque corresponds to vertical motion of input shaft and shocks into upper cover. The trajectory of the shaft centre of mass motion in case of the total radial clearances ±1 mm in the bearing is represented in Figure 3b. The trajectory parts beyond the solid line circle correspond to elastic deformation while shocks with amplitudes beyond the dotted line cause the unrecoverable plastic deformation of bearing elements (rings, rollers). The trajectory of shaft motion may be irregular and depends on many parameters, but the main factor is the radial clearance. That causes dynamic inclination of the shafts during the transient oscillations within the radial gaps, uneven distribution of instantaneous contact loads on the gears’ contacts and their failure.

The reasonable value of total clearance (about 1.6 mm) for undertaking maintenance actions can be determined from Figure 3c when recommended by bearing specification dynamic load capacity is almost reached.

The same representations of radial clearances effect on the dynamical loading can be built for the bolt studs following their cross-section and yield stress of material to determine a reasonable moment of their replacement taking into account the accumulated plastic deformation (number of tightening).

### 2.3. Algorithm of Vibration Signal Processing and Diagnostics

Maintenance staff, performing on-line vibration monitoring or scheduled manual measurements of the shafts supports in the gearboxes, establish the tendency of the main natural frequency, amplitude and phase changes. On this basis, they predict the wear of bearings and decide on the maintenance actions, namely, bolts tightening and bearing replacement, i.e., to serve the equipment by its real technical condition. The successful implementation of the proposed method of diagnostics into the maintenance practice supposes certain steps, which are described below:

(1) Determine the approximate range of natural shaft oscillations frequency in the supports by the model (in our case, the natural frequency is changing within the range 71–123 Hz).
(2) Determine the initial clearance in the bearing during its installation on the shaft with calibrated probes or take the value from the bearing specification.
(3) Perform vibration measurements on the bearing supports of transmission shafts and build the amplitude-frequency and phase-frequency diagrams.
(4) Determine by the measured vibration signal in the pre-calculated range the natural frequency *f*_n_ of the shaft oscillations and its higher harmonics (2 × *f*_n_, 3 × *f*_n_).
(5) Determine the change of natural frequency, the amplitude and phase at its higher harmonics by comparing values in the previous measurement and plot trend graphs.
(6) Determine the wear (radial gap) in the shaft bearing support by the small change of natural frequency, the amplitude and phase at the natural frequency and its higher harmonics (<10%).
(7) Determine the opening of the bolted joint by the significant decrease of natural frequency, amplitude and phase at the natural frequency and its higher harmonics (>10%).
(8) Carry out the tightening of the bolts in the bearing supports and continue vibration measurements.
(9) If the natural frequency, amplitude and phase at the natural frequency and its higher harmonics have not returned to the previous measurement values, replace the bolts and continue vibration measurements.
(10) If the natural frequency, amplitude and phase at the natural frequency and its higher harmonics have not returned to the previous measurement values, replace the bearing.
(11) Determine the final radial clearance of the bearing after its replacement and adjust, if necessary, the previously obtained relations of the natural frequency, amplitude and phase at the natural frequency and its higher harmonics by the measured gap size to increase the accuracy of diagnosis.

**Figure 4:**
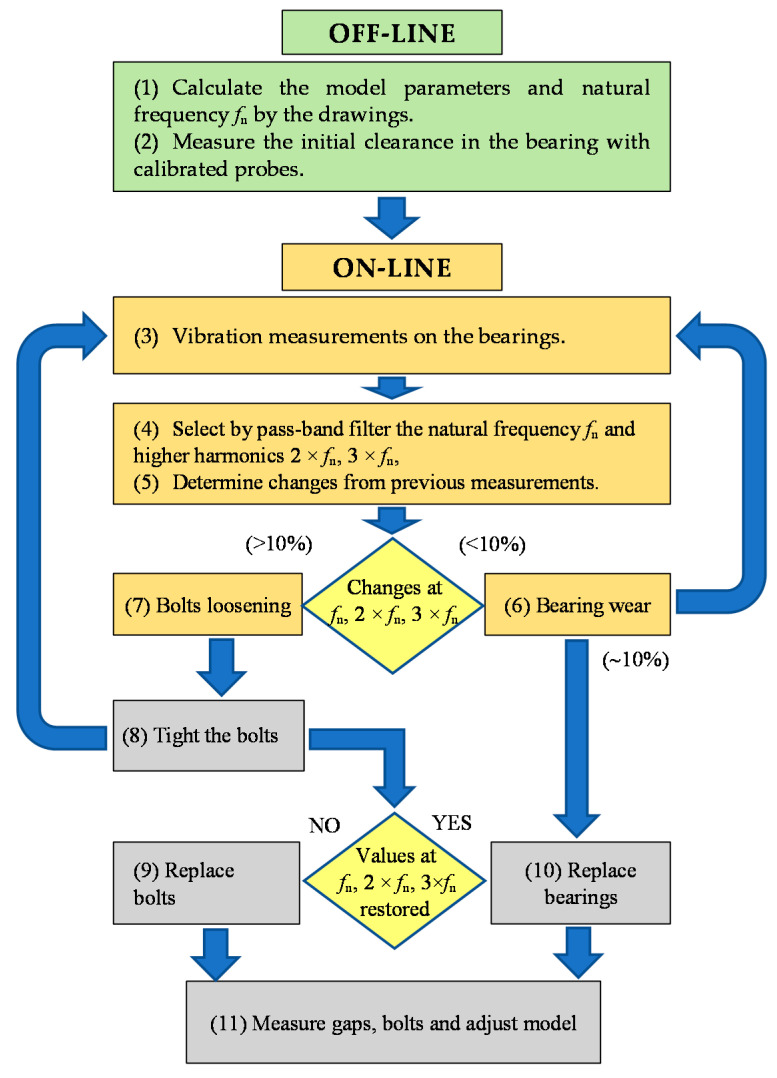
Algorithm of radial clearances and bolts loosening diagnostics.

The schematic representation of diagnostics algorithm is given in Figure 4, along with the corresponding numbers of the steps from the description.

The ISO Standard 10816 gives recommendations on ranges of absolute values of vibration velocity (mm/s) for the estimation of condition: good (0.28–1.8), satisfactory (2.8–4.5), unsatisfactory (7.1–11.2), and unacceptable (18.0–45.0) for Class III machines mounted on a large rigid foundation. However, these levels are only applicable to machines working under stationary loading and speed of the drive. The transition from between conditions corresponds to large differences in amplitude that can exceed 50%.

In heavy-duty gearboxes, comparing changes from a previous measurement is a more suitable approach. The difference in 10% for recognition between bearing clearance and bolt loosening is derived experimentally by signal observation. It is valid for the investigated class of heavy-duty gearboxes and recommended to maintenance staff who inspect drivelines and measure vibrations every 3–4 days because of a high risk of failures. In case of a permanent vibration monitoring system, this value can change by about 1.0–1.2% per day that corresponds to quick bolts loosening. After bolt tightening, vibration amplitudes are usually reduced by 8–10%. The wear of bearings and radial clearances contribute to gradual changes of the vibration amplitudes. For example, a change of natural frequency by 10% corresponds to a 20% stiffness change in bearings, which can develop over several months until replacement.

The abovementioned algorithm can be modified or adapted depending on maintenance practice at the certain industrial plant or machine type, e.g., when bolts and bearings are replaced separately. In this case, the algorithm is applied individually for every shaft support.

To implement the model-based diagnostics it is enough to have standard vibration sensors installed on the bearing supports of transmission shafts. Then, systematic measurements of vibration signals are conducted during the rising input load on the working tools of the machine and values of radial clearances with wear in bearings and bolted joints opening are determined. This is a principal distinction of the proposed method from the known approaches where a non-stationary (cyclically stationary) signal is interpreted as having a comparatively small deviation of average and dispersion in rotor velocity or loading. Instead, here the transient signal is used for diagnostic purposes and its spectral analysis is conducted in the range of natural frequencies but not the kinematic frequencies.

Since the natural frequencies of free oscillations are not changing during the repairs and replacement of shafts, the proposed method has a higher noise immunity compared to the known methods of diagnostics at kinematic bearing frequencies. For the recording and analysis of the transient vibration signals, triggering signals of mechanical torque in the transmission or electrical motor current (taking into account time delay of its response) can be used in condition monitoring systems.

## 3. Industrial Trials

An example of the implementation of the proposed method is given in this section. The method is tested in production conditions on a continuous hot rolling mill with the work rolls driven through the gearbox and the pinion stand.

Experiments are conducted using Industrial ICP^®^ accelerometers for permanent installation PCB 603CX1 (sensitivity 100 mV/g, amplitude range ±50 g) and line-powered signal conditioner PCB 482A22. Data is acquired by a National Instruments 16-ch 14-bit PCI-6071E A/D card and our software with the sampling frequency 2 kHz. The sensors were vertically mounted by screw stud on a magnetic base for curve surfaces PCB 080A133 (force 378 N) on the bearing supports of the input shaft of the gearbox. In parallel to vibration, the torsional load was measured on the motor shaft with the telemetry torque meter [104]. Angular clearances in the transmission were also measured by the method and device described in [74].

**Figure 5:**
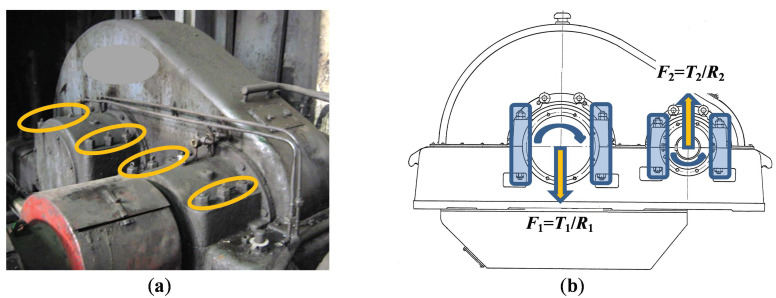
Industrial heavy-duty gearbox with bolt studs joints (**a**); scale drawing of bearing supports with applied torques and forces provoking shafts transient motions (**b**).

The investigated 1-stage gearbox is shown in Figure 5a. The bearings loading of gearbox shafts depends on gearbox design (helical or spur gears, power paths), shaft position over stages, the direction of shafts rotation concerning applied torques (see Figure 5b). The extremely high dynamic regimes of this gearbox working in the transmission of the industrial plant requires frequent (once per several days) maintenance actions for bolt stud tightening, especially on those bearing supports where shafts move from the bottom idle position to the upper position (the input shaft in our case).

Bolt stud joints of shafts are subjected to creep and elongation, although their nuts are fixed by second nuts on the top and with split-pins on the bottom. Sometimes, fixing nuts are welded to each other by rods. Bearings quickly deteriorate due to brinelling of rings and rollers under shock impacts from the gear shaft. Another problem is the contact load redistribution in gearbox couplings over the teeth length with subsequent cracks appearing at the end edges of teeth.

**Figure 6:**
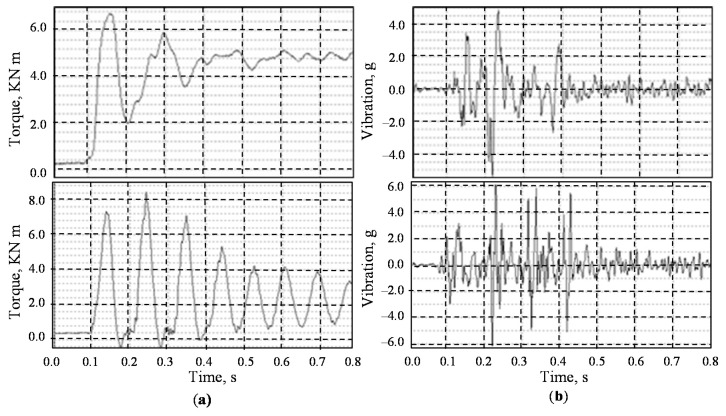
Measurements of torque in the motor shaft (**a**) and vertical vibration on the bearing supports (**b**) of two different gearboxes.

Several series of measurements were performed at intervals of 1 month at the same levels of technological load. Exemplary results of torque and vibration measurements conducted on the motor shafts of two gearboxes are shown in Figure 6. When analysing the transient processes in the gearboxes, it was found that the dynamic response of the shafts depends on the direction of their rotation. The maximum vibration is observed where the shaft gravity force and total force applied from gear coupling are acting in opposite directions. The torsional load pulse causes the shafts to move within the radial clearance during the transient mode of motion to the steady position.

Usually, the calculated natural frequencies of the shafts lateral vibration are higher (100–400 Hz) than the natural frequencies range of the torsional system (10–30 Hz). Therefore, the radial gaps in the bearings distort the torsional vibration signal by the high-frequency components near or slightly above the idle torque level. In case of minimal radial clearances, or when the shaft stays laying down in the bearing supports under load, the radial reaction to the torsional load repeats the patterns of the torque signal on the corresponding shaft.

### Diagnostic Parameters Calculations

**Figure 7:**
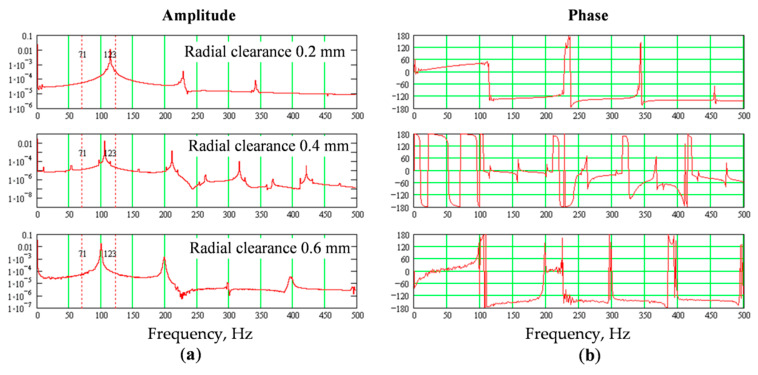
Amplitude (**a**) and phase (**b**) plots of shaft vibrations depending on the wear (radial clearance) in the bearing support without opening the bolted joints.

**Figure 8:**
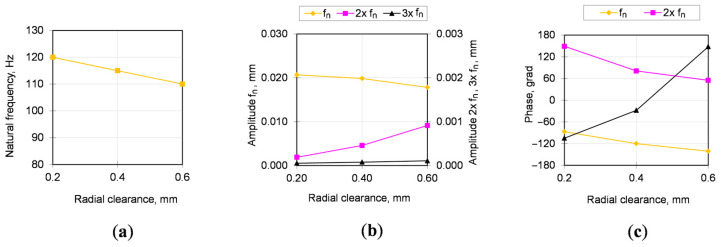
Natural frequency (**a**), amplitudes (**b**) and phases (**c**) of harmonics during transient vibrations of gearbox shaft without the bolted joint opening.

After each series of measurements, the amplitude-frequency and phase-frequency diagrams of the vibration signal were plotted in the low-frequency range of 500 Hz. Results of the measurement series No. 1 are shown in Figure 7 and Figure 8 and Table 2. The increase in the bearing wear (radial gaps) occurred from the minimum installation value (0.2 mm) to the maximum (0.6 mm). Graphs of the amplitude and phase diagrams of vibration with different wear (radial clearance) in the bearing without opening the bolted joint are shown in Figure 7.

The natural frequency in Figure 8 has a linear dependence on the gaps in the bearing support. The amplitude and phase of oscillations at the natural frequency and its higher harmonics are close enough to the linear dependence but have a different (proportional or inversely proportional) character on individual harmonics, which is used to increase the reliability of the method.

**Figure 9:**
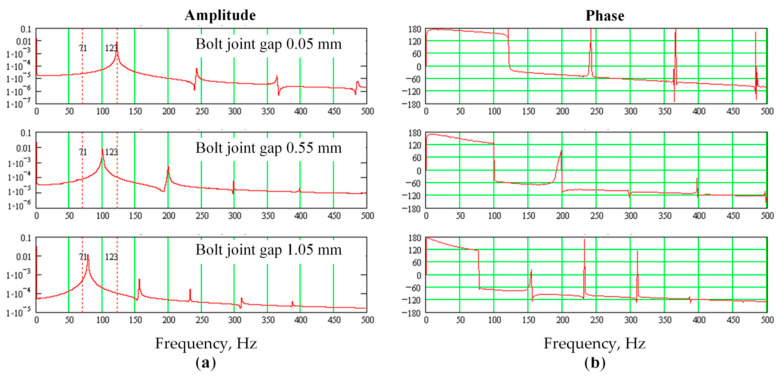
Amplitude (**a**) and phase (**b**) of shaft vibration depending on the opening of the bearing bolted joint.

**Figure 10:**
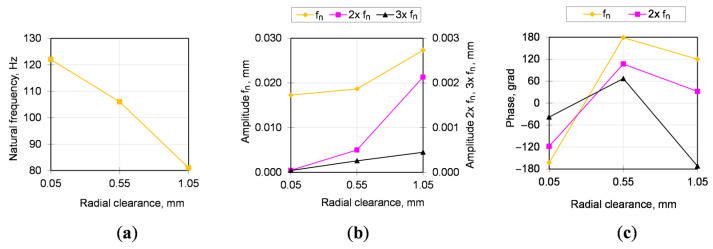
Natural frequency (**a**), amplitudes (**b**) and phases (**c**) of harmonics during transient vibrations of gearbox shaft with the wear of bolts (plastic elongation) and joint opening.

In the course of gearbox operation, the amplitude of the shaft oscillations gradually changed and the bolted joint opening increased from the minimum value (about 0.05 mm) to the maximum (1.05 mm) when bolts may break due to plastic elongation and loosening. Results of the measurement series No. 2 are shown in Figure 9 and Figure 10 and Table 3. In Figure 9 are graphs of the amplitudes and phases diagrams of vibration dependence on the wear (plastic elongation) of the bolts with the opening of the bearing bolted joint.

In measurement series of No. 1, the gap in the bearing was quite small (natural frequency about 123 Hz), but during the subsequent period of operation (see Figure 10) there were large changes in natural frequency, amplitude and phase of oscillations (greater than 10%) that indicated the deterioration of fastening bolts. The required tightening was performed, but in the next measurement No. 3 the natural frequency, amplitude and phase did not change to the values in the previous measurement, therefore the fastening bolts were completely replaced. The only available for measurement parameter after bolts replacement is their elongation, which was about 5%, i.e., above the elastic limit (plastic deformation) accumulated over the several times of their previous tightening.

Based on the experimental trials, the developed mathematical model is calibrated by the frequency of shaft free vibrations. For this gearbox with new gear shaft including new bearings and bolted studs, the natural frequency of shaft free vibrations is about 123–125 Hz.

To calibrate the dynamical model, the authors used manual adjustment of parameters without reverse estimation procedures used in multi-body dynamics.

This approach is justified by several factors:

- Only the main mode of shaft free vibrations is used for diagnostic purposes and, since the masses of components are exactly known from gearbox specifications, the stiffness is a single parameter for calibration.
- Frequencies of torsional and radial free vibrations are quite different and do not interfere in the spectrum, hence simple pass-band filtering is enough for their separation during the vibration signals analysis.
- The bearing stiffness changes slowly within months, but the bolted joint stiffness can change in several days or a week in our case; therefore, these parameters can be distinguished.

The opening of the bolted joints causes greater changes in natural frequency than the wear of the bearing, as well as the amplitude and phase at the natural frequency and its higher harmonics. In the period when the bearing support has a large gap, but the opening of the joint is not yet developed, the accuracy of its definition is not high. However, for up to 1 week (or earlier) from the start of the joint opening, the gap in the joint continues to increase faster than wear in the bearing and it can already be accurately diagnosed by the proposed method.

## 4. Discussion

Diagnostics of bearings clearances is most important for industrial plants and machines where the radial load or dynamic imbalance of shafts is comparable to their weight, i.e., shafts can move up and down inside the gaps from the initial idle position. The issue of diagnostics is that the gaps (opened part of radial clearances) of rolling bearings become closed when loading torque is applied to the driveline and they do not expose themselves in any way, although, they can increase torque amplification factor (TAF) up to 3–5 and cause bearing overloading.

In contrast to kinematic frequencies, natural frequencies are not changed with the speed of the drive (excepting high-speed turbines with journal sliding bearings). High amplitudes at these frequencies appear only during the torque increasing on the machines and reflect the wear of bearings. Therefore, the diagnostics of clearances can only be efficient during transient periods of loading.

The main natural frequency of shaft radial oscillation can be determined by the standard vibration sensors. Preliminary band-pass filtering can be applied in the range of shaft natural frequency pre-calculated by the dynamical model. There is a fairly stable and narrow range of natural frequencies, therefore, the automatic realization of this method may not be difficult in the data processing software.

In the case where one or more kinematic frequencies match the natural frequency range, the proposed method is difficult to implement, but it has an additional positive effect—plant personnel can determine potentially dangerous resonant oscillations in the gearboxes. This is a reason to change the operational speed of the machine or the design of the bearing support.

Angular clearances in the driveline always result in an increase of torsional dynamics and the appearance of high-frequency components in the signals of torque and vibration that can mask local defects in bearings or gear meshing. Therefore, signal recording for local defect detection (spalling, cracks) by the traditional methods should be carried out by logic triggering beyond the transient periods.

Radial clearances in the supports have a back effect on driveline system and change parameters of torsional vibrations (frequency, amplitude, and phase) even if the angular clearances are enough small or gaps are closed under load. Therefore, in heavy-duty gearboxes, when analysing vibration signals, it is necessary to account the ratio of the shafts gravity forces, shaft position in the gearbox stages, direction of rotation and torque magnitude when certain shaft can lose contact with the support during input load fluctuations.

## 5. Conclusions

According to the measurement results, the change of the shaft natural frequency of oscillations in the bearing support in the range of 71 … 123 Hz, spectrum amplitudes and phases at higher harmonics allow to diagnose radial clearance and fastening bolts loosening.

The shift of the natural frequency to smaller values corresponds to the lower stiffness of the bearing support with the greater opening of the joint or increased radial clearance (wear) in the bearing elements. The shift of the natural frequency to the greater values corresponds to the oscillation of the shaft at greater rigidity with less bolted joint opening and reduced radial gaps (wear) in the bearing elements. At the increased gaps and contact opening of a joint, oscillations occur on nonlinear sites of characteristics of the rigidity of support (pass through the first or second fracture), thus amplitudes and shift of phases on higher harmonics of natural frequency increase.

The proposed model-based approach provides a basis for on-line monitoring the total radial clearances in the bearings and the bolted joints loosening in the heavy-duty gearboxes of industrial plants. That allows scheduling maintenance actions (tightening or replacement) to prevent abrupt failures of machines.

Further research is aimed at the development of complex criterion of heavy-duty machine reliability during operation with the gradual wear of elements in their transmissions. This criterion (realized in a software procedure of condition monitoring systems) includes the accumulated cycles of loading and conditions of gears contact and teeth bending stress increasing under the action of torsional and lateral shafts oscillations in the bearing supports with radial clearances and potential bolt joint opening.

## Figures and Tables

**Table 1:** Classes, size ranges and mechanical properties of bolts in heavy-duty machines.

Class	Nominal Size Range, mm	Proof Load, MPa	Min. Yield Strength, MPa	Min. Tensile Strength, MPa
10.9	5–100	830	940	1040
12.9	1.6–100	970	1100	1220

**Table 2:** Results of vibration measurements (Figure 8).

Trial No	Clearance, mm	Natural Frequency Harmonics, Hz	Amplitude, mm	Phase, Grad
1	0.2	*f*_n_—120	0.020684	−87
	(measured)	2 × *f*_n_—240	0.000189	149
		3 × *f*_n_—360	0.000054	−105
2	0.4	*f*_n_—115	0.019850	−120
	(predicted)	2 × *f*_n_—230	0.000457	81
		3 × *f*_n_—345	0.000078	−28
3	0.6	*f*_n_—110	0.017818	−141
	(predicted)	2 × *f*_n_—220	0.000913	55
		3 × *f*_n_—330	0.000108	148

**Table 3:** Results of vibration measurements (Figure 10).

Trial No	Clearance, mm	Natural Frequency Harmonics, Hz	Amplitude, mm	Phase, Grad
1	0.05	*f*_n_—122	0.017270	−163
	(predicted)	2 × *f*_n_—244	0.000045	−118
		3 × *f*_n_—366	0.000041	−38
2	0.55	*f*_n_—106	0.018658	179
	(predicted)	2 × *f*_n_—212	0.000500	107
		3 × *f*_n_—321	0.000258	67
3	1.05	*f*_n_—81	0.027319	120
	(predicted)	2 × *f*_n_—162	0.002131	32
		3 × *f*_n_—243	0.000450	−172

## References

[1] Marciniszyn P.W. Maximizing Bearing Life through Proper Installation and Lubrication. Pumps & Systems. https://www.pumpsandsystems.com/maximizing-bearing-life-through-proper-installation-and-lubrication.

[2] Meyers K.E. Understanding Bearing Internal Clearance. https://www.machinedesign.com/motors-drives/article/21834658/understanding-bearing-internal-clearance.

[3] Bickford J. (1998). Handbook of Bolts and Bolted Joints.

[4] Krot P., Bobyr S., Dedik M. (2017). Simulation of backup rolls quenching with experimental study of deep cryogenic treatment. Int. J. Microstruct. Mater. Prop..

[5] Kraemer F., Klein M., Oechsner M. (2020). Fatigue strength of metric steel screws depending on pre-load and nut type. Eng. Fail. Anal..

[6] Liqin W., Li C., Dezhi Z., Le G. (2008). Nonlinear dynamics behaviors of a rotor roller bearing system with radial clearances and waviness considered. Chin. J. Aeronaut..

[7] Tiwari M., Gupta K., Prakash O. (2000). Effect of radial internal clearance of a ball bearing on the dynamics of a balanced horizontal rotor. J. Sound Vib..

[8] Kappaganthu K., Nataraj C. (2011). Nonlinear modeling and analysis of a rolling element bearing with a clearance. Commun. Nonlinear. Sci. Numer. Simul..

[9] Bartelmus W. (2014). Object and operation supported maintenance for mining equipment. Min. Sci..

[10] Bartelmus W., Chaari F., Zimroz R., Haddar M. (2010). Modelling of gearbox dynamics under time-varying nonstationary load for distributed fault detection and diagnosis. Eur. J. Mech. A Solids.

[11] Filatov A.A., Gartsman S.D., Zhukov A.A., Khrebin V.N. (2003). Determination of dynamic loads in pinion stands and reduction gears of rolling mills. Stal’.

[12] Korennoy V.V. (2015). Simulation of dynamic processes in gearboxes of rolling stands. Metall. Min. Ind..

[13] Krot P.V. Dynamics and Diagnostics of the Rolling Mills Drivelines with Non-Smooth Stiffness Characteristics. Proceedings of the 3rd International Conference on Nonlinear Dynamic, ND-KhPI2010.

[14] Krot P.V. (2019). Dynamical processes in a multi-motor gear drive of heavy slabbing mill. J. Vibroeng..

[15] Zhou S., Ren Z., Song G., Wen B. (2015). Dynamic characteristics analysis of the coupled lateral-torsional vibration with spur gear system. Int. J. Rotating Mach..

[16] Chen Y., Gao Q., Guan Z. (2017). Self-loosening failure analysis of bolt joints under vibration considering the tightening process. Shock Vib..

[17] Kasei S. (2007). A study of self-loosening of bolted joints due to repetition of small amount of slippage at bearing surface. J. Adv. Mech. Des. Syst. Manuf..

[18] Nassar S.A., Housari B.A. (2007). Study of the effect of hole clearance and thread fit on the self-loosening of threaded fasteners. J. Mech. Des..

[19] Brake M.R.W. (2018). The Mechanics of Jointed Structures. Recent Research and Open Challenges for Developing Predictive Models for Structural Dynamics.

[20] Zhou J., Liu J., Ouyang H., Cai Z., Peng J., Zhu M. (2019). Self-loosening behavior of bolted joints subjected to dynamic shear load. Int. J. Mod. Phys. B.

[21] Argatov I., Sevostianov I. (2010). Health monitoring of bolted joints via electrical conductivity measurements. Int. J. Eng. Sci..

[22] Pan Q., Pan R., Chang M., Shao C., Liu X., Xu X. Reliability evaluation of bolt fastening force based on ultrasonic measurement method. Proceedings of the IEEE International Conference on Mechatronics and Automation (ICMA).

[23] Pan Q., Pan R., Shao C., Chang M., Xu X. (2020). Research review of principles and methods for ultrasonic measurement of axial stress in bolts. Chin. J. Mech. Eng..

[24] Mba D. (2002). Applicability of acoustic emissions to monitoring the mechanical integrity of bolted structures in low speed rotating machinery: Case study. NDT E Int..

[25] Zhang Z., Liu M., Liao Y., Su Z., Xiao Y. (2018). Contact acoustic nonlinearity (CAN)-based continuous monitoring of bolt loosening: Hybrid use of high-order harmonics and spectral sidebands. Mech. Syst. Signal Process..

[26] Zhang Z., Liu M., Su Z., Xiao Y. (2017). Continuous monitoring of residual torque of loose bolt in a bolted joint. Procedia Eng..

[27] Wang F., Song G. (2020). Monitoring of multi-bolt connection looseness using a novel vibro-acoustic method. Nonlinear. Dyn..

[28] Zhang Z., Xu H., Liao Y., Su Z., Xiao Y. (2017). Vibro-acoustic modulation (VAM)-inspired structural integrity monitoring and its applications to bolted composite joints. Compos. Struct..

[29] Liu S., Li Y., Wang T., Luo Y. (2014). A piezoelectric active sensing method for detection of bolt load loss. Sens. Rev..

[30] Hosoya N., Niikura T., Hashimura S., Kajiwara I., Giorgio-Serchi F. (2020). Axial force measurement of the bolt/nut assemblies based on the bending mode shape frequency of the protruding thread part using ultrasonic modal analysis. Measurement.

[31] Shao J., Wang T., Yin H., Yang D., Li Y. (2016). Bolt looseness detection based on piezoelectric impedance frequency shift. Appl. Sci..

[32] Kędra R., Rucka M., Wahab M. (2020). Bolted joint monitoring using the elastic wave propagation. Lecture Notes in Mechanical Engineering, Proceedings of the 13th International Conference on Damage Assessment of Structures, Porto, Portugal, 9–10 July 2019.

[33] Wang F., Chen Z., Song G. (2020). Monitoring of multi-bolt connection looseness using entropy-based active sensing and genetic algorithm-based least square support vector machine. Mech. Syst. Signal Process..

[34] Wang C., Wang N., Ho S., Chen X., Pan M., Song G. (2018). Design of a novel wearable sensor device for real-time bolted joints health monitoring. IEEE Internet Things J..

[35] Chen D., Huo L., Song G. (2020). EMI based multi-bolt looseness detection using series/parallel multi-sensing technique. Smart Struct. Syst..

[36] Nazarko P., Ziemianski L. (2017). Force identification in bolts of flange connections for structural health monitoring and failure prevention. Procedia Struct. Integr..

[37] Huynh T.-C., Dang N.-L., Kim J.-T. (2018). Preload monitoring in bolted connection using piezoelectric-based smart interface. Sensors.

[38] Huynh T.-C., Ho D.-D., Dang N.-L., Kim J.-T. (2019). Sensitivity of piezoelectric-based smart interfaces to structural damage in bolted connections. Sensors.

[39] Dravid S., Tripathi K., Chouksey M. (2014). Role of washers in controlling loosening of full threaded bolted joints. Procedia Technol..

[40] Kong X., Li J. An image-based feature tracking approach for bolt loosening detection in steel connections. Proceedings of the SPIE 10598, Sensors and Smart Structures Technologies for Civil, Mechanical, and Aerospace Systems.

[41] Park J.-H., Huynh T.-C., Choi S.-H., Kim J. (2015). Vision-based technique for bolt-loosening detection in wind turbine tower. Wind Struct..

[42] Kong X., Li J. (2018). Image registration-based bolt loosening detection of steel joints. Sensors.

[43] Zhang Y., Sun X., Loh K.J., Su W., Xue Z., Zhao X. (2020). Autonomous bolt loosening detection using deep learning. Struct. Health Monit..

[44] Zhao X., Zhang Y., Wang N. (2019). Bolt loosening angle detection technology using deep learning. Struct. Control Health Monit..

[45] Pham H.C., Ta Q.-B., Kim J.-T., Ho D.-D., Tran X.-L., Huynh T.-C. (2020). Bolt-loosening monitoring framework using an image-based deep learning and graphical model. Sensors.

[46] Nguyen T.-C., Huynh T.-C., Ryu J.-Y., Park J.-H., Kim J.-T. Bolt-loosening identification of bolt connections by vision image-based technique. Proceedings of the SPIE 9804, Nondestructive Characterization and Monitoring of Advanced Materials, Aerospace, and Civil Infrastructure.

[47] Huynh T.-C., Park J.-H., Jung H.-J., Kim J.-T. (2019). Quasi-autonomous bolt-loosening detection method using vision-based deep learning and image processing. Autom. Constr..

[48] Cha Y.-J., You K., Choi W. (2016). Vision-based detection of loosened bolts using the Hough transform and support vector machines. Autom. Constr..

[49] Wang C., Wang N., Ho S.-C., Chen X., Song G. (2020). Design of a new vision-based method for the bolts looseness detection in flange connections. IEEE Trans. Ind. Electron..

[50] Xu C., Huang C.-C., Zhu W.-D. (2019). Bolt loosening detection in a jointed beam using empirical mode decomposition–based nonlinear system identification method. Int. J. Distrib. Sens. Netw..

[51] Zhang T., Biswal S., Wang Y. (2020). SHMnet: Condition assessment of bolted connection with beyond human-level performance. Struct. Heal. Monit..

[52] Wang T., Song G., Liu S., Xiao H. (2013). Review of bolted connection monitoring. Int. J. Distrib. Sens. Netw..

[53] Deka A., Rao A., Kamath S., Gaurav A., Gangadharan K.V., Dutta S., Inan E., Dwivedy S. (2020). Modeling and experimental studies on the dynamics of bolted joint structure: Comparison of three vibration-based techniques for structural health monitoring. Advances in Rotor Dynamics, Control, and Structural Health Monitoring. Lecture Notes in Mechanical Engineering.

[54] Anginthaya K., Kuchibhatla S.A.R., Gangadharan K.V., Kishan A. (2018). A comparative study on the effectiveness of system parameters in monitoring pre-load loss in bolted joints. J. Braz. Soc. Mech. Sci. Eng..

[55] Wang Z., Liu M., Zhu Z., Qu Y., Wei Q., Zhou Z., Tan Y., Yu Z., Yang F. (2019). Clamp looseness detection using modal strain estimated from FBG based operational modal analysis. Measurement.

[56] You R., Ren L., Song G. (2020). A Novel comparative study of European, Chinese and American codes on bolt tightening sequence using smart bolts. Int. J. Steel Struct..

[57] Todd M.D., Nichols J.M., Nichols C.J., Virgin L.N. (2004). An assessment of modal property effectiveness in detecting bolted joint degradation: Theory and experiment. J. Sound Vib..

[58] Celic D., Boltezar M. (2008). Identification of the dynamic properties of joints using frequency response functions. J. Sound Vib..

[59] Milanese A., Marzocca P., Nichols J., Seaver M., Trickey S.T. (2008). Modeling and detection of joint loosening using output-only broad-band vibration data. Struct. Health Monit..

[60] Tol S. (2012). Dynamic Modeling of Structural Joints. Master’s Thesis.

[61] Hu Y.-J., Guo W.-G., Jiang C., Zhou Y.-L., Zhu W. (2018). Looseness localization for bolted joints using Bayesian operational modal analysis and modal strain energy. Adv. Mech. Eng..

[62] Wang J., Xu M., Zhang C., Huang B., Gu F. (2020). Online bearing clearance monitoring based on an accurate vibration analysis. Energies.

[63] Xu M., Feng G., He Q., Gu F., Ball A. (2020). Vibration characteristics of rolling element bearings with different radial clearances for condition monitoring of wind turbine. Appl. Sci..

[64] Yakout M., Nassef M.G.A., Backar S. (2019). Effect of clearances in rolling element bearings on their dynamic performance, quality and operating life. J. Mech. Sci. Technol..

[65] Rehab I., Tian X., Gu F., Ball A.D. (2018). The influence of rolling bearing clearances on diagnostic signatures based on a numerical simulation and experimental evaluation. Int. J. Hydromechatron..

[66] Tol S., Ozguven H.N. (2015). Dynamic characterization of bolted joints using FRF decoupling and optimization. Mech. Syst. Signal Process..

[67] Ren T.Q., Liu Z.R., Xu X.D., Liu Y., Wang X.D. (2019). Clearance measurement equipment for gas lubricated dynamic pressure bearing of gyro motor. Int. J. Precis Eng. Manuf..

[68] Karlberg M. (2010). Approximated stiffness coefficients in rotor systems supported by bearings with clearance. Int. J. Rotating. Mach..

[69] Kostek R., Landowski B., Muslewski L. (2015). Simulation of rolling bearing vibration in diagnostics. Vibroeng. Procedia.

[70] Rabeyee K., Tang X., Xu Y., Zhen D., Gu F., Ball A.D. Diagnosing the change in the internal clearances of rolling element bearings based on vibration signatures. Proceedings of the 24th International Conference on Automation and Computing (ICAC).

[71] Liu J., Ding S., Wang L., Li H., Xu J. (2020). Effect of the bearing clearance on vibrations of a double-row planetary gear system. Proc. Inst. Mech. Eng. Part K J. Multi Body Dyn..

[72] Georgiadis A., Gong X., Meier N. (2018). Vibration analysis based on the spectrum kurtosis for adjustment and monitoring of ball bearing radial clearance. MATEC Web Conf..

[73] Omar R., Abdul Rani M.N., Yunus M.A. (2020). Representation of bolted joints in a structure using finite element modelling and model updating. J. Mech. Eng. Sci..

[74] Krot P. (2008). Methods and instrumentation for measuring wear in drivelines of rolling mills. Metall. Processes Equip..

[75] Kumar A., Jaiswal H., Ahmad F., Patil P.P. (2014). Dynamic vibration characteristics analysis of truck transmission gearbox casing with fixed constraint of vehicle frame based on FEA. Procedia Eng..

[76] Xianlong H., Tianli S. (2019). A new identification method for bolt looseness in wind turbine towers. Shock Vib..

[77] Nguyen T.C., Huynh T.-C., Yi J.-H., Kim J. (2017). Hybrid bolt-loosening detection in wind turbine tower structures by vibration and impedance responses. Wind Struct..

[78] Guoda C., Yang C., Yijie C., Shiming J. (2020). Dynamics modeling and experimental modal analysis of bolt loosening for lightning rod. J. Vibroeng..

[79] Brons M., Thomsen J.J., Sah S.M., Tcherniak D., Fidlin A., Kerschen G., Brake M., Renson L. (2020). Analysis of transient vibrations for estimating bolted joint tightness. Nonlinear Structures and Systems: Conference Proceedings of the Society for Experimental Mechanics Series, Proceedings of the 37th Conference and Exposition on Structural Dynamics (IMAC 2019), Orlando, FL, USA, 28–31 January 2019.

[80] Sah S.M., Thomsen J.J., Brons M., Fidlin A., Tcherniak D. (2018). Estimating bolt tightness using transverse natural frequencies. J. Sound Vib..

[81] Brons M., Thomsen J.J., Sah S.M., Tcherniak D., Fidlin A. (2018). Estimating bolt tension from vibrations: Transient features, nonlinearity, and signal processing. Mech. Syst. Signal Process..

[82] Kelin A., Larin O., Naryzhna R., Trubayev O., Vodka O., Shapovalova M. Estimation of residual life-time of pumping units of electric power stations. Proceedings of the IEEE 14th International Conference on Computer Sciences and Information Technologies (CSIT).

[83] Sah S.M., Thomsen J.J., Fidlin A. (2020). Transverse vibrations of tightened bolts: Simplified modeling of tension-dependent boundary stiffness and damping. Eng. Struct..

[84] Li Q., Jing X. (2021). A novel second-order output spectrum based local tuning method for locating bolt-loosening faults. Mech. Syst. Signal Process..

[85] Miao R., Shen R., Zhang S., Xue S. (2020). A Review of bolt tightening force measurement and loosening detection. Sensors.

[86] Guarino J., Hamilton R., Fischer W. (2009). Acoustic detection of bolt detorquing in structures. Proc. Meet. Acoust..

[87] Bruand G., Chatelain F., Granjon P., Martin N., Duret C. (2020). Reconstructing shaft orbit using angle measurement to detect bearing faults. Mech. Syst. Signal Process..

[88] Zhang H., Borghesani P., Smith W.A., Randall R.B., Shahriar M.R., Peng Z. (2021). Tracking the natural evolution of bearing spall size using cyclic natural frequency perturbations in vibration signals. Mech. Syst. Signal Process..

[89] Pnevmatikos N.G., Blachowski B., Hatzigeorgiou G.D., Swiercz A. (2016). Wavelet analysis based damage localization in steel frames with bolted connections. Smart Struct. Syst..

[90] Gelman L., Patel T.H. (2015). Novel rolling bearing diagnosis technology using spectral kurtosis and the wavelet higher-order spectra. Insight Non Destr. Test. Cond. Monit..

[91] Gelman L., Murray B., Patel T.H., Thomson A. (2013). Diagnosis of bearings by novel non-linear non-stationary higher order spectra. Insight Non Destr. Test. Cond. Monit..

[92] Gelman L., Murray B., Patel T.H., Thomson A. (2014). Vibration diagnostics of rolling bearings by novel nonlinear non-stationary wavelet bicoherence technology. Eng. Struct..

[93] Ciszewski T., Gelman L., Swedrowski L. (2016). Current-based higher-order spectral covariance as a bearing diagnostic feature for induction motors. Insight Non Destr. Test. Cond. Monit..

[94] Bartelmus W., Zimroz R. (2009). A new feature for monitoring the condition of gearboxes in non-stationary operating conditions. Mech. Syst. Signal Process..

[95] Obuchowski J., Wylomanska A., Zimroz R. (2014). Recent developments in vibration based diagnostics of gear and bearings used in belt conveyors. Appl. Mech. Mater..

[96] Wodecki J., Michalak A., Zimroz R. (2018). Optimal filter design with progressive genetic algorithm for local damage detection in rolling bearings. Mech. Syst. Signal Process..

[97] Wodecki J., Michalak A., Zimroz R. (2021). Local damage detection based on vibration data analysis in the presence of Gaussian and heavy-tailed impulsive noise. Measurement.

[98] Hebda-Sobkowicz J., Zimroz R., Pitera M., Wyłomańska A. (2020). Informative frequency band selection in the presence of non-Gaussian noise—A novel approach based on the conditional variance statistic with application to bearing fault diagnosis. Mech. Syst. Signal Process..

[99] Wodecki J., Kruczek P., Bartkowiak A., Zimroz R., Wyłomańska A. (2019). Novel method of informative frequency band selection for vibration signal using Nonnegative Matrix Factorization of spectrogram matrix. Mech. Syst. Signal Process..

[100] Wodecki J., Michalak A., Zimroz R., Barszcz Y., Wyłomańska A. (2019). Impulsive source separation using combination of Nonnegative Matrix Factorization of bi-frequency map, spatial denoising and Monte Carlo simulation. Mech. Syst. Signal Process..

[101] Krot P., Zimroz R., Michalak A., Wodecki J., Ogonowski S., Drozda M., Jach M. (2020). Development and verification of the diagnostic model of the sieving screen. Shock Vib..

[102] Bolshakov P.V., Krot V.V., Verenev A.P., Dalichuk A.P., Korennoy V.V. (2004). Application of Non-Stationary Operating Modes of Rolling Mills for Diagnostics of Clearances. Fundamental and Applied Problems of Ferrous Metallurgy: Collection of Scientific Works. Institute of Ferrous Metallurgy of the National Academy of Sciences of Ukraine. http://dspace.nbuv.gov.ua/handle/123456789/21489.

[103] Krot P., Prykhodko I., Raznosilin V., Zimroz R. (2020). Model based monitoring of dynamic loads and remaining useful life prediction in rolling mills and heavy machinery. Advances in Asset Management and Condition Monitoring.

[104] Krot P.V. (2008). Telemetering systems for monitoring dynamic loads in the drive lines of rolling mills. Vib. Mach. Meas. Reduct. Prot..

